# Amino Acid-Derived Supramolecular Assembly and Soft Materials

**DOI:** 10.3390/molecules29194705

**Published:** 2024-10-04

**Authors:** Shuaishuai Nie, He Zhao, Jiayi Sun, Qingtao Liu, Yongming Cui, Wen Li

**Affiliations:** 1 State Key Laboratory of Supramolecular Structure and Materials, College of Chemistry, Jilin University, Changchun 130012, China; niess1320@mails.jlu.edu.cn (S.N.); hezhao23@mails.jlu.edu.cn (H.Z.); jiayis23@mails.jlu.edu.cn (J.S.); 2 National Local Joint Engineering Laboratory for Advanced Textile Processing and Clean Production, Wuhan Textile University, Wuhan 430200, China; cuiyongming@wtu.edu.cn

**Keywords:** amino acids, supramolecular assembly, nanostructures, soft materials

## Abstract

Amino acids (AAs), serving as the primary monomer of peptides and proteins, are widely present in nature. Benefiting from their inherent advantages, such as chemical diversity, low cost, ease of modification, chirality, biosafety, and bio-absorbability, AAs have been extensively exploited to create self-assembled nanostructures and supramolecular soft materials. In this review article, we systematically describe the recent progress regarding amino acid-derived assembly and functional soft materials. A brief background and several classified assemblies of AAs and their derivatives (chemically modified AAs) are summarized. The key non-covalent interactions to drive the assembly of AAs are emphasized based on the reported systems of self-assembled and co-assembled AAs. We discuss the molecular design of AAs and the general rules behind the hierarchical nanostructures. The resulting soft materials with interesting properties and potential applications are demonstrated. The conclusion and remarks on AA-based supramolecular assemblies are also presented from the viewpoint of chemistry, materials, and bio-applications.

## 1. Introduction

Biomolecular assembly has aroused considerable research interests, due to its recognized importance in the fabrication of hierarchical nanostructures and biomaterials toward various applications [1,2,3]. Typical examples include DNA- [4], protein- [5], and peptide-based supramolecular assemblies [6]. Among them, peptides are one of the unique building blocks, owing to their rich chemical diversity and diverse biofunctions [7,8]. Specifically, the short oligopeptides (below 10 amino acid residues), consisting of relatively simple structures, hold promising potential in the development of cost-efficient biomaterials from the viewpoint of application [9]. For this purpose, ultra-short oligopeptides (less than 3 amino acid residues) have been intensively designed, and their predictable secondary structures have been leveraged to construct controllable nanostructures and bioactive soft materials [10,11]. As the primary monomer of peptides, amino acids (AAs) are minimalistic segments and also ideal candidates for the construction of biomaterials due to their inherent biocompatibility, bio-absorbability, low cost, and ease of production [12,13]. However, most of the natural AAs (except aromatic AAs) cannot form ordered molecular packing in solution because the intermolecular affinity among the natural AAs is normally insufficient to rival the solvation effect, leading to association failure in solution [12]. To improve the assembling ability of AAs, two parallel strategies have been developed. One is the co-assembly between AAs and other chemical species [14,15], which greatly unlocks the potential of AAs in the development of AA-based supramolecular nanostructures and soft materials because a broad catalogue of organic and inorganic species can be combined with AAs via very flexible non-covalent interactions. Another is the chemical modification of natural AAs by conjugating aromatic or aliphatic segments with AAs at either the N- or the C-terminus [16,17,18]. The resulting AA derivatives show strong intermolecular affinity and stacking propensity in solutions, ensuring the formation of thermodynamically stable assemblies.

**Figure 1:**
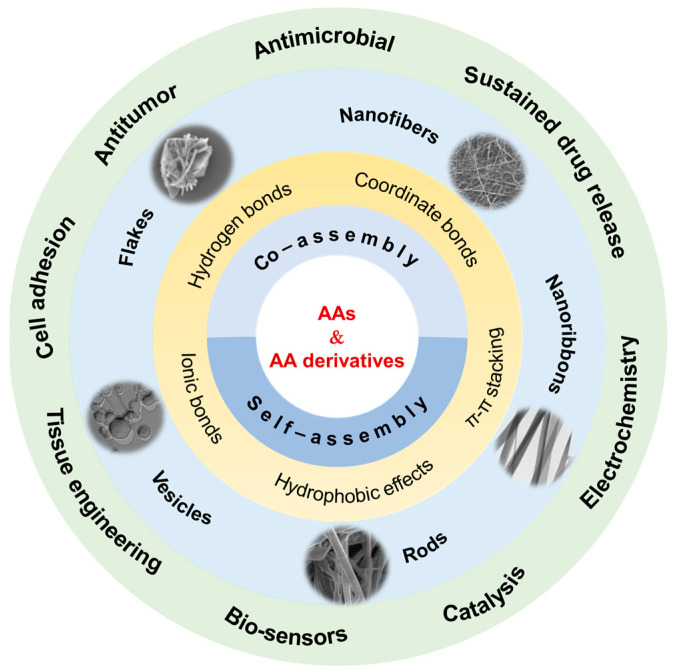
Schematic illustration of amino acid (AA)-derived supramolecular assembly, nanostructures, and soft materials for diverse applications.

Over the past two decades, a great deal of research has been pursued to fabricate AA-based supramolecular assemblies with different architectures, including spherical vesicles and droplets, one-dimensional (1D) rods, fibers, ribbons, tubes, two-dimensional (2D) flakes and plates, and three-dimensional (3D) networks. On the structural basis of the assemblies, emphasis has been placed on the driving forces and their synergistic effect, the chiral characters, the dynamic features, the value-added properties, and potential applications. This article offers a comprehensive overview of the recent advances in the development of single AA-derived supramolecular assembly and soft materials. Systems containing two or more AAs are out of the scope of this review, but we encourage interested readers to consult the related literature [19,20,21,22,23,24]. The basic issues we address are shown in Figure 1. We will classify the systems into three parts. The first part covers the natural AA assemblies, in which the natural AAs with aromatic or alkaline residues have the ability to form stable assemblies via self-assembly or co-assembly. The second part involves the self-assembly of the most widely studied AA derivatives. We describe the influence of hydrophobicity, volume fraction, and the topology of the substituents on the self-assembly behavior of AA derivatives. The third part is related to the co-assembly of AA derivatives. Diverse combinations of AA derivatives and chemical species will be demonstrated. This review focuses on the driving forces, assembled structures, dynamic responsiveness, and advances in functional soft materials.

## 2. Supramolecular Assembly of Natural Amino Acids

### 2.1. Self-Assembly of Natural Amino Acids

**Figure 2:**
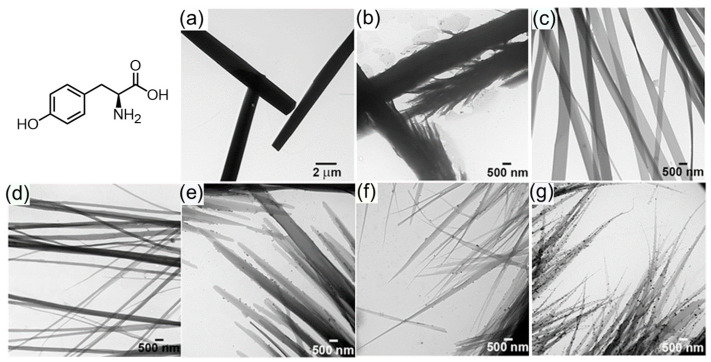
The schematic structure of Tyr and the concentration-dependent TEM images obtained from aqueous solution: (**a**) 1 mg/mL, (**b**) 750 μg/mL, (**c**) 500 μg/mL, (**d**) 250 μg/mL, (**e**) 150 μg/mL, (**f**) 100 μg/mL, (**g**) 50 μg/mL. Reproduced with permission from Ref. [26]. Copyright 2015 Wiley-VCH.

In fact, different microcrystalline structures have been reported based on the self-assembly of alkaline, acidic, and aliphatic AAs at dried state, but most of them tend to disassemble in an aqueous solution suffering from weak intermolecular interactions [25]. AAs with aromatic residues showed low solubility in an aqueous solution, suggesting their strong assembly propensity [26,27,28]. Ménard-Moyon and coworkers reported the aqueous self-assembly of tyrosine (Tyr) [26], which can form nanoribbons, nanofibers, and hydrogels in a wide range of concentrations driven by hydrophobic interactions and π-stacking of the side chain together with the hydrogen bonds and ionic bonds of the zwitterionic main chain of Try (Figure 2). In another work, fluorescent nanotubes were obtained from the self-assembly of Try and tryptophan (Trp) in an ethanol solution [27], demonstrating that the self-assembly nanostructures are strongly related to the solvent environments.

**Figure 3:**
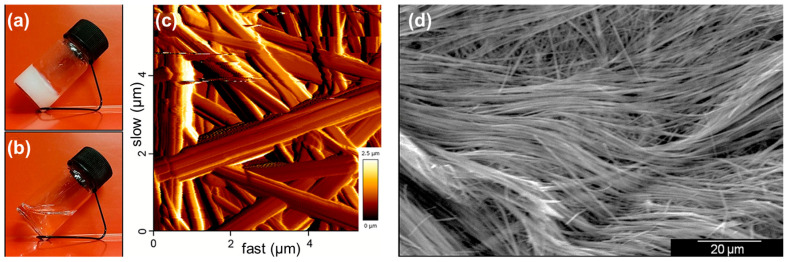
Thermo-reversible hydrogel of Phe (**a**) below and (**b**) above the critical gelation temperature. (**c**) AFM and (**d**) SEM images of dried hydrogel of Phe, highlighting the entanglement of a fibrous network with widths ranging from 200 to 900 nm. Reproduced with permission from Ref. [29]. Copyright 2020 Wiley-VCH.

Aromatic *L*-phenylalanine (*L*-Phe) can also self-assemble into hydrogel with fibrous networks [28]. More interestingly, the gel-to-crystal transformation process was observed in this system. Although the authors proposed that the electrostatic interaction and hydrogen bonding between NH_3_^+^ and COO^−^ drove the formation of hydrogel, the π-π stacking and hydrophobic effect of the aromatic group cannot be ignored. Ramalhete et al. found that Phe can form thermo-reversible hydrogels (Figure 3) over a wide range of concentrations [29]. Furthermore, the addition of Ser to Phe led to the formation of weaker materials, while the addition of Trp tended to stabilize the three-dimensional network structure of Phe, indicating the importance of π-π stacking and the hydrophobic effect. Chirality-dependent self-assembly of Phe has been reported by Singh and colleagues. They observed that the mixture of *D*-Phe and *L*-Phe can greatly inhibit the 1D growth, giving rise to the formation of sheet-like structure [30]. With this finding in mind, the authors proposed that *D*-Phe could be used as a therapeutic molecule for phenylketonuria, which is known to be associated with the toxicity of fibers formed by *L*-Phe.

**Figure 4:**
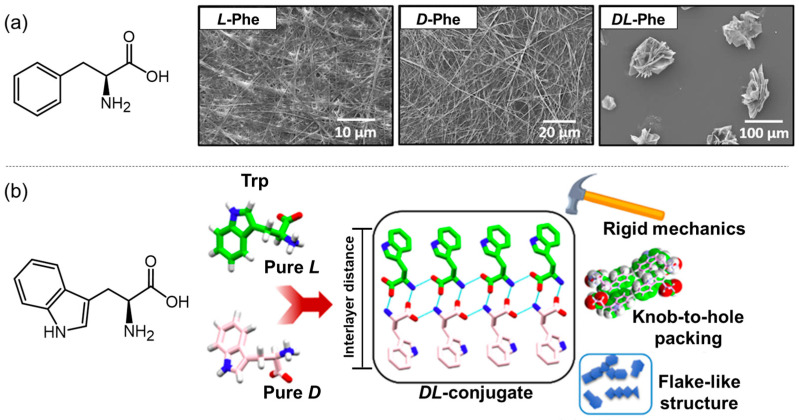
(**a**) The schematic structure of Phe and the SEM images of the enantiomeric and the racemic Phe; (**b**) the schematic structure of Trp and the single-crystal structures of the racemic Trp. Reproduced with permission from Ref. [31]. Copyright 2020 American Chemical Society.

Similar chirality dependence was observed in the case of Trp and Phe [31]. Taking Phe as an example, the pure enantiomers slowly self-assembled into 1D nanofibers in water (Figure 4a). However, the *DL*-mixed systems rapidly formed crystalline flake-like structures. The same phenomenon was also observed in the case of Trp. X-ray single-crystal structure analysis revealed that the *L*-Trp formed ordered molecular arrangement via head-to-tail H-bonding and edge-to-face π−π stacking. However, the racemic counterparts adopted head-to-tail H-bonding and face-to-face π−π stacking with the centrosymmetric dimer unit (Figure 4b). The authors proposed that this perfect “knobs-in-holes” fitting of aromatic surfaces through face-to-face arrangement is the dominant factor in stabilizing the flake-like structure of the racemic AAs. The same group further explored the synergistic assembly between Phe and other AAs [32]. Based on the interlayer separation distances (Figure 4b) observed in the X-ray single-crystal structure, the authors classified the natural AAs into three different subgroups. They found that Phe can synergistically assemble with isoleucine (Ile) or methionine (Met) possessing similar interlayer space to that of Phe. Consequently, spherical (Phe/Ile) or flake-like (Phe/Met) nanostructures were observed, which were different from that of single-component assemblies. However, a self-sorting assembly was observed in the mixed systems of Phe with glycine (Gly) and alanine (Ala), respectively, owing to the space-mismatched effect between Phe and Gly or Ala. Anand et al. investigated the synergistic assembly of Phe, Tyr, and Trp under simulated physiological conditions [33]. Transmission electron microscopy (TEM) images revealed the formation of hybrid nanofibrils with an amyloid structure. Molecular dynamic simulations indicated that the nanofibrils with a hydrophobic exterior and hydrophilic interior were mainly driven by hydrogen bonding, π−π stacking, and CH-π interactions. Furthermore, the hybrid nanofibers can induce protein cross-seeding.

### 2.2. Co-Assembly of Natural Amino Acids and Organic Species

**Figure 5:**
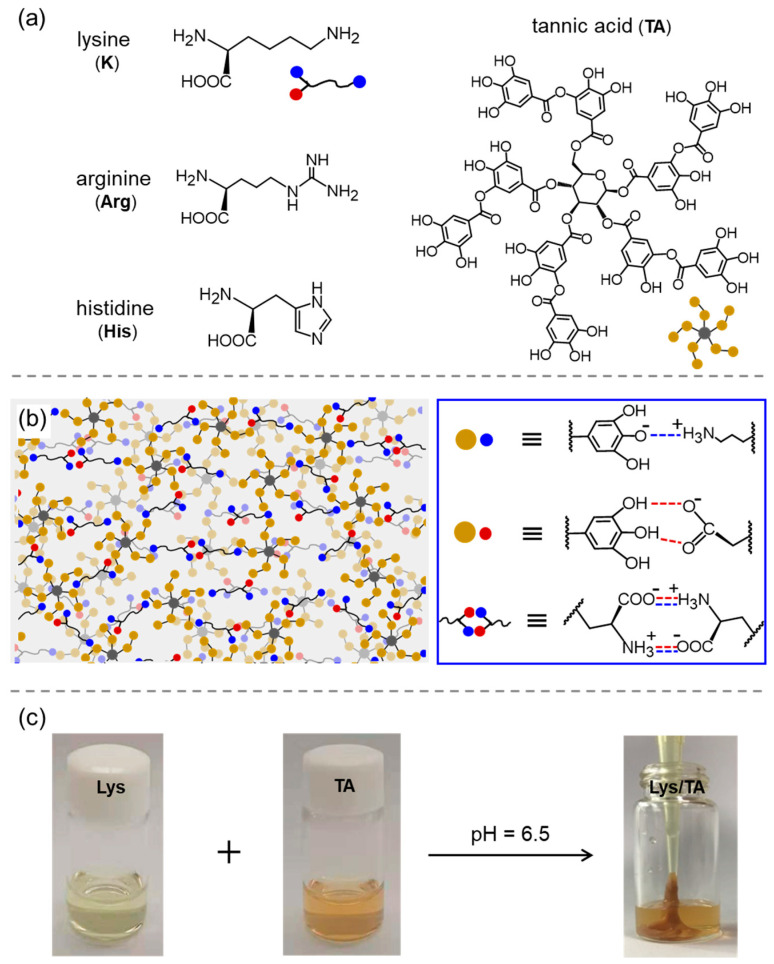
(**a**) Schematic drawing of the alkaline AAs and tannic acid. (**b**) Schematic illustration of the co-assembly between TA and alkaline AAs. (**c**) Photographs of the preparation process of TA/Lys adhesives. Reproduced with permission from Ref. [12]. Copyright 2023 American Chemical Society.

In early studies, several groups investigated the co-assembly of arginine and saturated or unsaturated fatty soaps via hydrophobic interactions, ionic bonds, and the unique bidentate hydrogen bonds between the carboxylate and guanidinium [34,35,36,37,38,39]. It was observed that arginine cannot only stabilize the micelle or vesicle structures of the fatty soaps but also regulate the structural transformation from the vesicle to the sponge or lamellar phase [34,35,36]. In addition, the assembled structures showed a stimulus responsibility to pH, temperature, and glycerol [38,39]. Recently, Meng et al. reported a co-assembled system based on tannic acid (TA) and alkaline AAs (Figure 5a), such as lysine (Lys), arginine (Arg), and histidine (His) [12]. Detailed studies demonstrated that TA carrying multiple phenol groups can interact with the mainchain and side chains of the alkaline AAs through ionic and hydrogen bonds, which were helpful for the formation of macroscopic materials with crosslinked networks (Figure 5b). The obtained soft materials (Figure 5c) showed underwater adhesion, self-repair, and shear thinning characters. A thermosensitive hydrogel has been developed based on Lys or glutamic acid (Glu), chitosan, and αβ-glycerophosphate. The co-assembled system gelled rapidly at 37 °C, and thus, could be used for sustained release of a model drug [40]. Hu et al. observed that the introduction of β-Ala into the chitosan/poly-(γ-glutamic acid) hydrogel can enhance the structural stability and other physical properties [41]. β-Ala allows the hydrogel to be pH responsive and can release more benzalkonium chlorides in an alkaline environment, serving as a potential alkaline wound dressing material.

### 2.3. Assembly of Natural Amino Acids/Metal Ion Complexes

**Figure 6:**
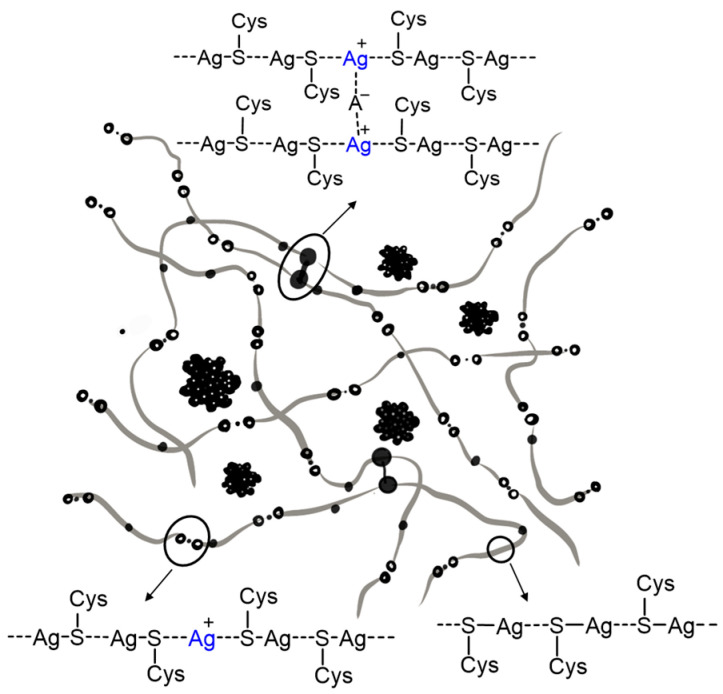
Schematic drawing of the network structure of supramolecular hydrogel based on the co-assembly of cysteine (Cys) and silver nitrate. Reproduced with permission from Ref. [42]. Copyright 2014 Pleiades Publishing.

AAs can interact with diverse metal ions, forming ordered nanostructures and soft materials. Pakhomov and coworkers developed a supramolecular hydrogel based on the co-assembly of cysteine (Cys) and silver nitrate [14]. It was observed that Cys and silver ion first formed a silver mercaptide complex in an aqueous solution, which further maturated into chain-like supramolecular oligomers via the charge transfer interaction between sulfur (donor) and silver (acceptor). Thereafter, the chain-like oligomers interacted with each other in the existence of Na_2_SO_4_ to form a hydrogel via the possible salt bridge–hydrogen bonds between the zwitterionic main chains of adjacent Cys molecules (Figure 6). TEM images showed that the hydrogel possessed a “curly hair” structure. The co-assembled metallo-hydrogel exhibited potent antimicrobial activities against both Gram-positive and Gram-negative bacteria [42]. The same metallo-hydrogel was also constructed by Vishnevetskii and colleagues. TEM revealed the existence of a network matrix that resulted from interconnected nanofibers with silver nanoparticles attaching to the network nodes. The hydrogel effectively inhibited the proliferation of MCF-7 breast cancer cells but showed negligible toxicity to embryonic fibroblast WI-38 cells [43].

A pH and thermal responsible metallo-hydrogel was generated based on the co-assembly of His and zinc ion [44]. It was observed that the His and zinc ions can form the [Zn(His) (H_2_O)_3_(OH)] complex followed by stacking into supramolecular polymer chains, nanofibers, and network structures. Chirality-dependent gelation was observed and the gelation ability was proportional to the enantiomer excess percentage (ee%). Along this line, Phe-Cu(II) supramolecular metallogel was developed. Field emission scanning electron microscopy (FE-SEM) images revealed that the metallogel consisted of sheet-like structures [45]. Density function theory calculation indicated that the 2:1 coordination stoichiometry of Cu(II) to Phe enabled the complex [Cu(Phe)_2_] to adopt an optimized planar geometry with hydrophobic phenyl rings forming an open pattern, which facilitated the ordered molecular stacking. Again, the co-assembled complex [Cu(Phe)_2_] showed chirality-dependent gelation behavior. Decreasing the enantiomeric excess percentage (ee%) suppressed the gelation ability of the Phe-Cu(II) system. Similarly, a co-assembled L-Phe/Zn^2+^ hydrogel was successfully constructed in an alkaline solution [46]. Energy dispersive spectrum, fourier transform infrared (FT-IR) spectroscopy, ultraviolet–visible (UV-vis) spectroscopy, and small-angle x-ray scattering analyses confirmed that L-Phe molecules can pack together via π-π stacking, while the carboxylate group of L-Phe can coordinate with Zn^2+^. As a result, a three-dimensional network consisting of rod-like structures with a length of ten micrometers was observed.

**Figure 7:**
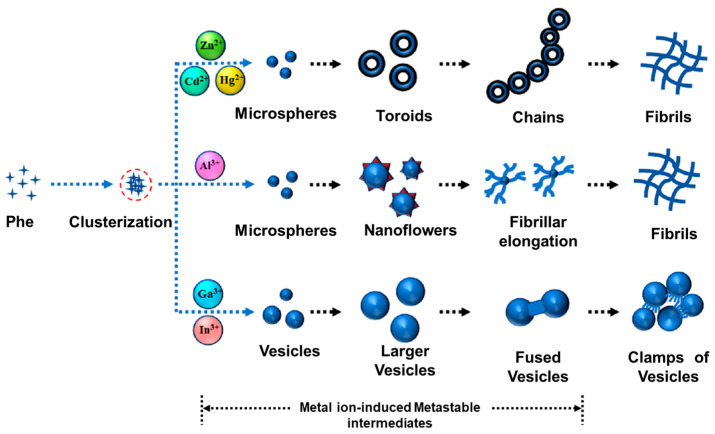
Evolution of the self-assembly of Phe in the presence of various divalent and trivalent metal ions. Reproduced with permission from Ref. [48]. Copyright 2022 American Chemical Society.

The influence of metal ions with different charges and sizes on the assembled structure of Phe and Trp was investigated by Bachi and coworkers [47]. The Na^+^ (pKa ~ 14), Mg^2+^, and Ca^2+^ (pKa ~ 11–12) ions have no significant effect on the fibrous assemblies of aromatic AAs. In contrast, the ions with lower pKa values such as Zn^2+^ (pKa ~ 9), Al^3+^ (pKa ~ 5), and Ga^3+^ (pKa ~ 3) can inhibit the formation of fibrous structures, accompanied with enhanced fluorescent emission. The authors suggested that the coordination between the main chain of Phe or Trp with the high-nuclear metal ions can reduce the aggregation-induced quenching effect via weakening of the molecular stacking, giving rise to the enhanced emission. The self-assembly behavior of Phe in the presence of different metal ions was further investigated [48]. As shown in Figure 7, Phe molecules can co-assemble with divalent metal ions (Zn^2+^, Cd^2+^, and Hg^2+^) to form spherical structures at the initial stage, which fuse into toroid intermediate and, finally, form fibrils. Trivalent Al^3+^ and Phe can co-assemble into nanoflower intermediate and, finally, longer fibrils. In the case of Ga^3+^ and In^3+^, vesicular intermediate and final clamps of vesicles were observed. The authors showed that the hydrophobic interactions among the side chain of Phe and the coordination bonds between Phe and metal ions dominated the co-assembled structures at the initial stage, the Phe–solvent interactions, hydrogen bonding, and π-π stacking induced the formation of different intermediates and the final structures.

These kinds of co-assembled systems were further extended to various hydrophobic AAs (such as Phe, Leucine (Leu), and Trp) and metal ions (such as Cu^2+^, Cd^2+^, Co^2+^, Ni^2+^, Pb^2+^, Zn^2+^, Hg^2+^, La^3+^, Sm^3+^, and Lu^3+^) [49]. In some cases, the hydrophobic effect of the side chains of the AAs and the coordination interactions between the AAs and the metal ions can drive the formation of insoluble Pickering foams in alanine aqueous solutions. AAs and Cd^2+^ or Zn^2+^ co-assembled into needle-like structures, while plate-like structures with larger sizes were observed in the case of Co^2+^ and Pb^2+^. The needle-like structures possessed higher efficiency for stabilizing Pickering foams. Interestingly, the foaming and defoaming can be achieved by adjusting the pH value. Wei and coworkers investigated the co-assembly of AAs (such as Gly, Ala, Phe, Trp, Tyr, and Arg) and metal ions (Ag^+^, Cu^2+^, and Co^2+^) [50]. TEM images revealed that Ag^+^ can induce the AAs to form zero-dimensional (0D), one-dimensional (1D), and three-dimensional (3D) superstructures. While AA/Cu^2+^ tended to form sheet-like superstructures, hedgehog superstructures, 2D flakes, and 1D fibers were observed in the case of the AA/Co^2+^ system. All these co-assembled materials exhibited excellent photocatalytic properties.

### 2.4. Co-Assembly of Natural Amino Acids and Inorganic Nanoclusters

**Figure 8:**
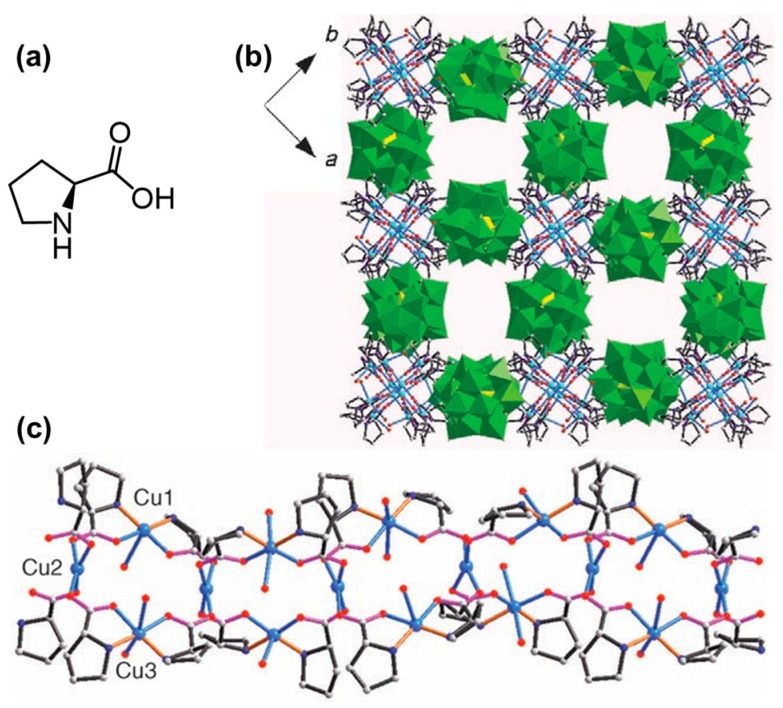
(**a**) Schematic structure of Pro; (**b**) the 3D single-crystal structure of Pro/Cu^2+^/[BW_12_O_40_]^5−^ (B yellow, C gray, N dark blue, O red, Cu blue, W green); (**c**) the 2D stacking structure of Pro/Cu^2+^/[BW_12_O_40_]^5-^ (C gray, N dark blue, O red, Cu blue). Reproduced with permission from Ref. [61]. Copyright 2006 Wiley-VCH.

Apart from metal ions, inorganic nanoclusters are another kind of candidate for co-assembling with AAs. It was observed that the Phe- or citrate-capped Au clusters (Phe@Au or Cit@Au) can function as a catalyst to induce the assembly of aromatic AAs [15]. As a result, Phe formed unique fibrils in the presence of Phe@Au, while it relatively fused fibrils in the presence of Cit@Au. His formed a thick dendritic morphology in the presence of Phe@Au, while a fibril morphology in the presence of Cit@Au. Interestingly, Trp showed similar dendritic structures when co-assembled with both Phe@Au and Cit@Au. These results indicated that the gold nanoparticles may affect the intermolecular forces between metabolites, thereby facilitating the formation of stable suprastructures. Polyoxometalates (POMs), consisting of an early transition metal (W, Mo, or V) bridged by oxygen, are a class of inorganic nano-sized clusters with precise chemical structures, various compositions, reversible redox activities, catalysis, photo- or electrochromic properties, and negative charges [51,52]. Much earlier, ionic co-assembly between cationic proteins or peptides and anionic POMs had already been reported here and there [53,54,55,56,57,58]. Cindrić et al. report an example of an AA/POM co-assembled system based on Gly-coordinated molybdovanadate complex K_2_[HMo_6_^VI^V^V^O_22_(Gly)_3_]·8H_2_O [59]. In a subsequent work, co-assembled hybrid microspheres were constructed using carboxyl-protected (methyl ester) Phe and Keggin-type [PW_11_O_39_{Sn(C_6_H_4_)CuC(C_6_H_4_)COOH}]^4-^ [60]. Molecular dynamics simulations showed that hydrophobic effects and π-π interactions drove the assembly. Chiral three-dimensional open frameworks with helical channels (Figure 8) were reported by An et al. using pure proline (Pro) enantiomers, Cu^2+^, and Keggin-type [BW_12_O_40_]^5-^ [61]. The chiral transfer from Pro ligands to the POM cluster was achieved via the copper cations as a central bridge. Additionally, the authors developed another kind of three-dimensional framework by replacing Pro with Gly [62]. Subsequently, this kind of ionic [HGly]_3_[PW_12_O_40_] and [HGly]_3_[PMo_12_O_40_] complexes were explored as highly efficient catalysts for the oxidation of cyclohexene by hydrogen peroxide [63]. Similar enhancement in catalysis has also been observed in the case of ((Gly)_3_HAl(H_2_O)VW_11_O_39_, (Gly)_3_H_2_Cr(H_2_O)VW_11_O_39_, (Gly)_3_HZn(H_2_O)VW_11_O_39_, and (Gly)_3_H_3_Cr(H_2_O)FeW_11_O_39_ [64]. It was concluded that the glycine played an important role in improving the stability and specific surface area of the POMs. Following these reports, a series of co-assembled complexes have been constructed for photoreduction and enantioselective catalysis [65,66,67,68,69].

**Figure 9:**
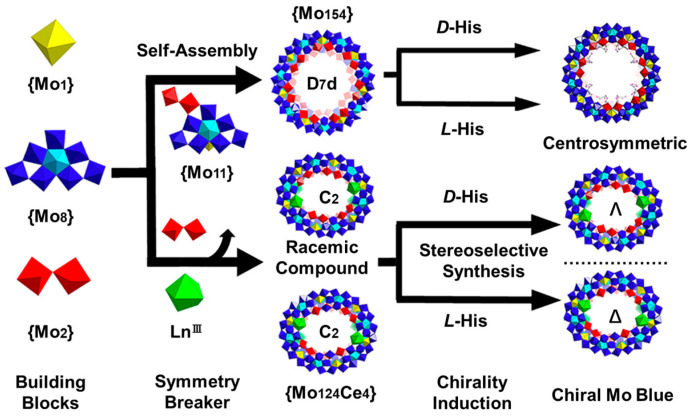
Schematic co-assembly route of enantiomeric His, Ln^3+^, and Mo clusters. Reproduced with permission from Ref. [70]. Copyright 2019 American Chemical Society.

Chiral Mo Blue wheels (Figure 9) were synthesized based on POM, lanthanide ions, and AAs [70]. In particular, His/{Mo_124_Ce_4_} can be used as a closed reaction vessel to, in situ, generate chiral {Mo_8_} and selectively oxidize tryptophan. These chiral molybdenum blue wheels showed potential in asymmetric catalysis. Mirzaei et al. prepared a series of AA/POM co-assemblies, most of which existed in the form of binary AAs [71]. The authors demonstrated that there were significant hydrogen bonds and van der Waals forces between AAs and the O atoms located at the surface of the POM. Farhadipour et al. constructed several co-assembled nanotubes with lengths of a few micrometers and diameters of about 20 to 200 nm based on AAs and POMs, such as ((Ile)_3_PMo_12_O_40_, (Ile)_3_PW_12_O_40_, (Cys)_3_PMo_12_O_40_, and (Cys)_3_PW_12_O_40_ [72]. In another work, vesicular structures have been constructed based on weakly-type [EuW_10_O_36_]^9−^ and natural AAs (Arg, Lys, His, Glu, aspartic acid (Asp), Leu, Ala, and Phe) via electrostatic and hydrophobic interactions [73]. It was observed that basic AAs (Arg, Lys, and His) tended to enhance the luminescence of [EuW_10_O_36_]^9−^ in an aqueous solution (2-fold improvement in the fluorescence intensity), while acidic AAs (Glu, Asp) inhibited the luminescence of [EuW_10_O_36_]^9−^, and the nonpolar AAs (Leu, Ala, and Phe) had no effect on the luminescence of [EuW_10_O_36_]^9−^. Following the co-assembly strategy, coordination polymers with good thermal and watery stability, and proton conduction (4.97 × 10^−3^ S cm^−1^) were prepared based on Gly, lanthanide ions (La^3+^ or Ce^3+^), and (Cr(OH)_6_Mo_6_O_18_ [74]. In another example, Lys/POM nanoparticles were incorporated into the ultrathin polyethylene films via the layer-by-layer technique for improving the stability of the film and offering the intrinsic electrochemical properties of POM [75]. Using the same technique, the co-assembled Gly/PMo_12_ nanoparticles were also introduced into a polyelectrolyte capsule with the aim of developing nanoreactors or a drug delivery system [76]. Daima et al. fabricated a kind of ternary system based on Tyr, silver nanoparticles (AgNPs), and POMs. AgNPs were modified by Tyr to form Tyr/AgNPs, followed by covering H_3_PW_12_O_40_ and H_3_PMo_12_O_40_ to form Tyr/AgNP ternary composites with enhanced antimicrobial activity [77]. Recently, co-assembled complexes, consisting of [PTi_2_W_10_O_40_]^7−^ and different AAs (Gly, Pro, Cys, Glu, Phe, Ala, Val, Ser, Thr), have been utilized to inhibit HIV-1 [78].

**Figure 10:**
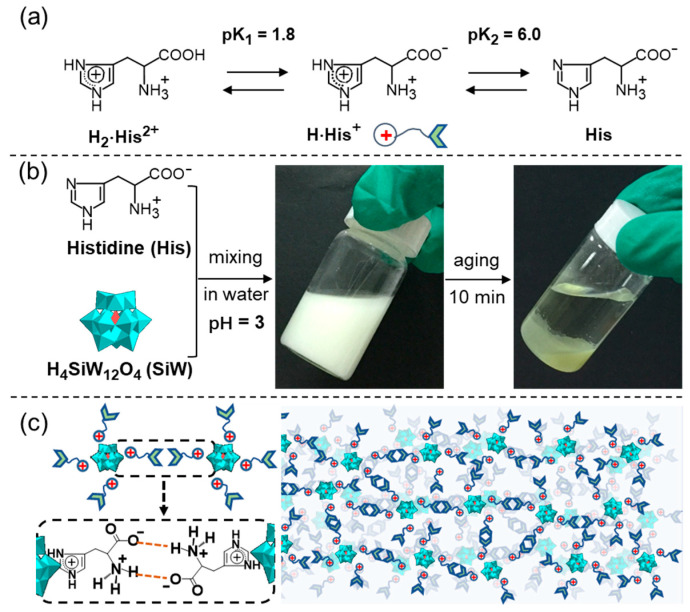
(**a**) Protonated species of His present in aqueous solutions at different pH values; (**b**) chemical structures of His and H_4_SiW_12_O_40_ (SiW), and their co-assembly process in water; (**c**) packing model of His/SiW coacervate. Reproduced with permission from Ref. [79]. Copyright 2017 Wiley-VCH.

Taking advantage of the protonated side chain of His, Xu and coworkers reported an unexpected complex coacervate based on the co-assembly of His and H_4_SiW_12_O_40_ (SiW) in an aqueous solution [79]. As illustrated in Figure 10, the His/SiW coacervates resulting from the liquid–liquid phase separation (LLPS) were driven by the ionic and hydrogen bonds between the protonated imidazole group of His and anionic SiW, together with the salt bridge–hydrogen bonds among the zwitterionic main chain of His. Similar complex coacervates could also be produced by mixing other alkaline AAs and POMs by carefully controlling the cationic side chains and the zwitterionic main chains of the alkaline AAs. Microcalorimetry measurements demonstrated that both enthalpy and entropy drove the coacervation [80]. Thus, the co-assembled coacervates were susceptible to temperature, resulting in the upper critical solution temperature (UCST) phenomenon. The UCST of the coacervate was proportional to the molar concentration of the AAs. A low pH (pH < 5.5) can increase the viscosity of the coacervate and lead to the formation of a gel-like phase. Inspired by this observation, functional supramolecular hydrogels with fibrous networks were obtained when the anionic POMs simultaneously interacted with the protonated α−NH_2_ and the protonated side chains of alkaline AAs in a strongly acidic solution [81]. More interestingly, the resulting hydrogels showed both UV-responsive photochromism and NIR-responsive gel–sol transition.

**Figure 11:**
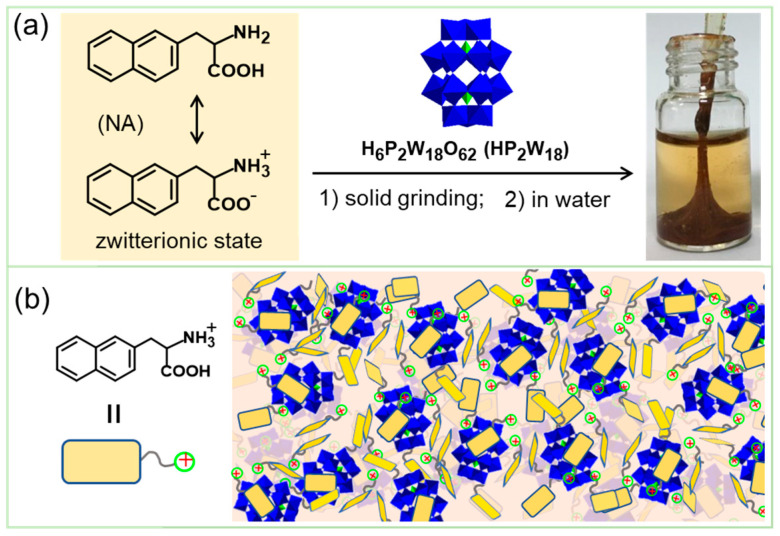
(**a**) Chemical structures of 3-(2-naphthyl)-l-alanine (NA) and H_6_P_2_W_18_O_62_, their co-assembly process in water; (**b**) schematic packing model of NA/H_6_P_2_W_18_O_62_ adhesive. Reproduced with permission from Ref. [82]. Copyright 2018 Wiley-VCH.

In a subsequent work, biomimetic underwater adhesives (Figure 11a) were created via the co-assembly of aromatic AAs and heteropolyacids (HPAs) in an acidic solution [82]. In this case, the ionic bonds and charge transfer interactions between aromatic AAs and HPAs, and the hydrophobic and π-π interactions among the aromatic groups of the AAs were identified to play a significant role in stabilizing the network structures of the formed adhesives even under the water line (Figure 11b). More interestingly, the reversible electrochromism and redox property of the HPAs remained, which enabled the co-assembled adhesives to be a processable electrode coating for the fabrication of self-powered electrochromic batteries [82] and flexible supercapacitors [83,84].

## 3. Self-Assembly of Amino Acid Derivatives

### 3.1. Self-Assembly of Aromatic Ring-Modified Amino Acid Derivatives

**Figure 12:**
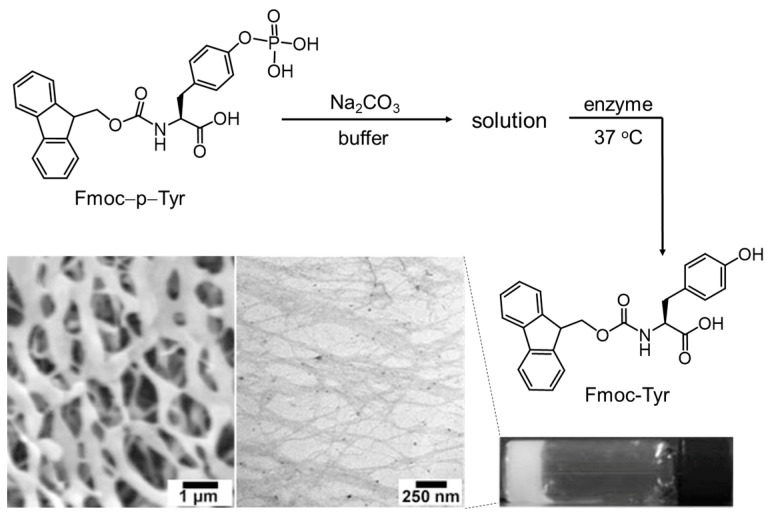
Schematic drawing of the transformation from Fmoc-p-Tyr to Fmoc-Tyr, and the SEM and TEM images of the Fmoc-Tyr hydrogel. Reproduced with permission from Ref. [85]. Copyright 2004 Wiley-VCH.

Chemically coupling aromatic units with natural AAs is an alternative method for the creation of AA-based nanostructures and functional soft materials. Yang and coworkers observed that 9-fluorenylmethyloxycarbonyl-modified Tyr (Fmoc−Tyr) can self-assemble into stable hydrogel, while the phospholated Fmoc-Tyr (Fmoc−p−Tyr) remained in sol state under the same condition [85]. As a consequence, the authors designed a kind of enzyme-responsive hydrogel (Figure 12) with the aid of a dephosphorylation enzyme. A similar phenomenon was observed by Sahoo et al. [86]. Sasselli et al. simulated the self-assembly behavior of Fmoc-Tyr by optimizing the CHARMM force field parameters [87]. The simulation results also supported the fibrous structures of Fmoc-Tyr. Apart from Fmoc-Tyr, Fmoc-Phe can also self-assemble into a stable hydrogel with long fibrillar aggregates driven by non-covalent interactions, such as hydrophobic interactions and π-stacking of aromatic rings, hydrogen bonds, and/or ionic bonds of the main chains [17,88]. This kind of Fmoc-Phe nanofiber was explored as a host to fabricate fluorescent silver nanoclusters [16]. It was illustrated that the carboxyl groups of Fmoc-Phe was favorable for the reduction in the Ag^I^ ions in the existence of light. As a result, ultra-small Ag_4_ nanoclusters (<2 nm) were formed in situ and trapped in the matrix of the gel phase. Meanwhile, the morphology of the Fmoc-Phe gel changed from nanofiber to nanovesicle. These Ag nanoclusters are stable and can even be stored at 4 ^º^C for more than 4 months without the loss of any fluorescence properties. To extract the importance of hydrogen bonding in promoting the 1D assembly of Fmoc-Phe, Rajbhandary et al. designed and synthesized the corresponding peptoid (Fmoc-Nphe), where the *α*-NH_2_ of Fmoc-glycine (Fmoc-Gly) was substituted by the phenyl group [89]. It was shown that Fmoc-Nphe tended to form 2D plate-like structures driven by intramolecular π−π interactions owing to the lack of directional hydrogen bonding interactions in the carbamate portion. A similar phenomenon was observed in several Fmoc-Phe objects with different substituents on the phenyl ring.

**Figure 13:**
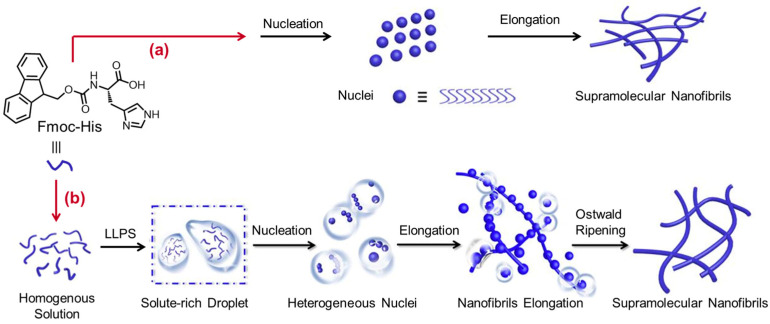
(**a**) The conventional nucleation and elongation route. (**b**) Schematic illustration of the formation of self-assembling Fmoc-His nanofibrils via the liquid–liquid phase separation (LLPS) dominates the nucleation and elongation route. In contrast with the traditional nucleation–elongation mechanism, the LLPS into solute-rich and solute-poor liquid phases is a critical step prior to the nucleation of supramolecular nanofibrils based on amphiphilic amino acid self-assembly. The solute-rich liquid droplets act as nucleation precursors and the hydrated nanoclusters within them serve as the nucleation loci. Finally, the thermodynamically favorable nanofibrils elongate from the metastable intermediates proceeding by Ostwald’s step rule. Reproduced with permission from Ref. [97]. Copyright 2019 Wiley-VCH.

Apart from the hydrogen bond, the steric effect of the residue can also affect the self-assembly behavior of Fmoc-Phe. For example, the introduction of a single halogen atom (X) into the residue of Fmoc-Phe can significantly promote self-assembly due to the complex balance between electronic and steric effects [90]. With this finding in mind, Ryan and colleagues conjugated the Fmoc unit at the N-terminus of fluorine-substituted Phe (F_5_-Phe) [91]. The resulting Fmoc-F_5_-Phe showed rapid assembly and gelation even at low concentrations. The aforementioned results suggest that the self-assembly process and the properties of AA derivates could be mediated by controlling the steric hindrance, π–π interactions, and hydrophobic effect. In another report, Duraisamy et al. found that the Fmoc-*β*-Phe molecules can undergo a rearrangement from J- to H-like aggregates, causing a transition from a scaffold-like morphology to an entangled fibrillar network [92]. The resulting hydrogel showed a shape memory property. Xie et al. observed that the Fmoc-Trp hydrogel consisted of rigid and orderly arranged nanofibers, while Fmoc-modified methionine (Fmoc-Met) and Fmoc-Tyr hydrogels were comprised of flexible nanofibers [93]. The structural difference was closely related to the antibacterial activity. As such, the resulting hydrogels showed antibacterial activity against Gram-positive bacteria as follows: Fmoc-Trp > Fmoc-Met > Fmoc-Tyr. These results suggest that the rigidity and orderliness of the nanofibers can be utilized to control the antibacterial activity of the hydrogel. Notably, all the hydrogels showed almost no antibacterial activity against Gram-negative bacteria, possibly due to the different cell wall structure between Gram-negative and -positive bacteria. Draper et al. found that the strategy of the Fmoc-assisted assembly of AAs can be extended to Fmoc-Met, Fmoc-Gly, and Fmoc-Ile [94]. All of the Fmoc-AAs can form self-supporting hydrogels with fibrous structures under pH modulation by glucono-δ-lactone. However, Fmoc-Phe and Fmoc-Tyr showed a strong propensity to form single crystals. The authors found that Fmoc-Phe showed the same molecular arrangements both in the crystal state and in the gel state. In contrast, different molecular arrangements were observed in the case of Fmoc-Tyr. Molecular stacking of planar Fmoc moieties dominated the formation of the crystalline structure, whereas the fibrous structures in the gel phase were mainly driven by hydrogen bonding. Reddy et al. proposed that the gelation of the Fmoc-AAs resulted from an appropriate balance among aromatic π-π stacking, hydrogen bonding, ionic bonds, and the hydrophobic effect [95]. When the N- and C-terminus of Asp were modified by Fmoc, the resulting Asp derivative can also form a stable hydrogel with entangled fibrils [96]. In particular, the hydrogel showed the ability to complex with calcium ions and to induce mineralization, which conversely improved the osteogenic ability of the hydrogel. Yuan and coworkers investigated the self-assembly mechanism of Fmoc-AAs [97]. In contrast to the traditional nucleation–elongation mechanism, the authors demonstrated that the LLPS of Fmoc-AAs into solute-rich and solute-poor liquid phases was a critical step prior to the nucleation of supramolecular nanofibrils. In detail, the resulting solute-rich liquid droplets can act as nucleation precursors and the hydrated nanoclusters within them serve as the nucleation loci (Figure 13). Finally, the thermodynamic favorable nanofibrils elongated from the metastable intermediates proceeding by Ostwald’s step rule. In fact, the hydrophobic interactions governed the occurrence of LLPS, while hydrogen bonding and π-π stacking interactions predominated the dynamic evolution from droplets to nanofibrils. These findings shed light on the fundamental mechanism and provided an alternative way to control the self-assembly of AA derivatives.

**Figure 14:**
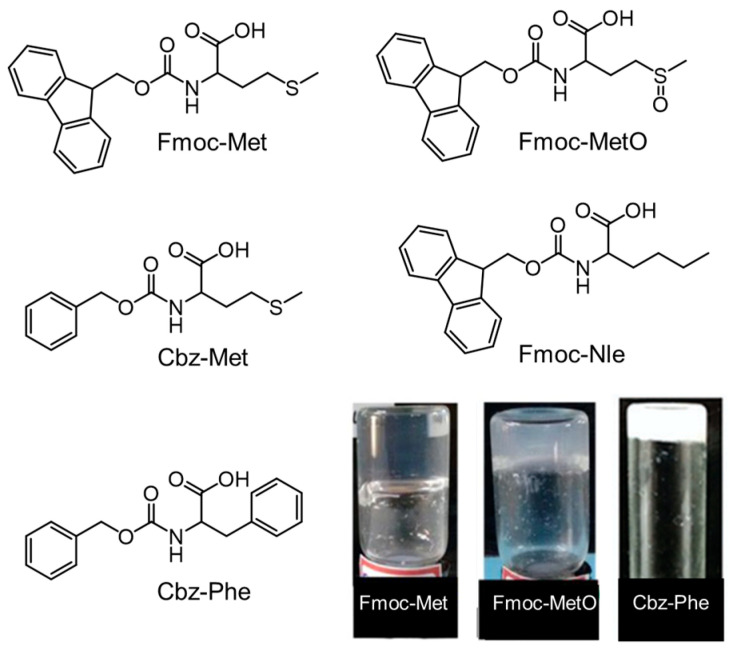
Schematic structures of Fmoc-Met (0.8 mM), Cbz-Met, Cbz-Phe (6.7 mM), Fmoc-MetO (8.0 mM), and Fmoc-Nle, and the photographs of the hydrogels resulting from the self-assembly of Fmoc-Met, Fmoc-MetO, and Cbz-Phe, respectively. The molecules Fmoc-Met, Cbz-Met, Fmoc-Nle, and Fmoc-MetO come from Ref. [95]. Cbz-Phe comes from Ref. [100]. Reproduced with permission from Refs. [95,100]. Copyright 2016 Wiley-VCH and 2020 Royal Society of Chemistry, respectively.

Beyond Fmoc moieties, different aromatic segments, such as naphthalene (Nap), benzoyloxy carbonyl (Cbz), pyrene (Pyr), and ferrocene (Fc), have been utilized to modify the natural AAs. Using the 4-NO_2_-Phe-based screening approach, Abraham et al. identified that the coupling Nap with 4-NO_2_-Phe could promote its gelation as efficiently as the Fmoc moiety [98]. Reddy et al. found that Fmoc-Met can form a thermally reversible hydrogel (Figure 14), while benzoyloxy carbonyl methionine (Cbz-Met) cannot form a stable gel [95]. With this knowledge in mind, the authors proposed that the gelation resulted from an appropriate balance among aromatic π-π stacking, hydrogen bonding, ionic bonds, and the hydrophobic effect by comparing the self-assembly behavior of Fmoc-Met, oxidized Fmoc-Met (Fmoc-MetO), and Fmoc-modified norleucine (Fmoc-NIe). Increasing the hydrophobicity from Cbz-Met to Cbz-Phe, a stable hydrogel could be observed, and effective encapsulation and controllable release of drugs were achieved via dynamic aromatic interactions and hydrogen bonds [99]. Following these findings, a Cbz-Phe-based hydrogel with pH-responsiveness was explored [100]. In an acidic solution, the molecules can form a gel-like phase with fibril structures. The resulting hydrogel transformed into fluid sol via an autocatalytic reaction between urease and urea, which produced NH_3_ to induce the pH increase and dissociation of carboxylic dimer, leading to defibrillation and gel collapse. The addition of the acidic solution of urea resulted in the reformation of a self-supporting hydrogel, which showed a similar fuel-dissipative nature. With a further increase in the hydrophobicity in the case of N-[(phenyl methoxy) carbonyl]-L-tryptophan (Cbz-Trp), a hydrogel can be quickly observed at a weak acidity pH with the assistance of gluconolactone [101]. The resulting hydrogel exhibited good antimicrobial activity against *Escherichia coli* (*E. coli*) and *Staphylococcus aureus* (*S. aureus*). Similarly, N-(4-nitrobenzoyl)-Phe can self-assemble into an antimicrobial hydrogel in phosphate buffer saline driven by hydrogen bond interactions, hydrophobic interactions, and π–π stacking [102]. The gelation capacities of several Phe derivatives were compared in detail by Shi and coworkers by coupling fluorenyl, naphthyl, naphthalenoxyl, and cinnamoyl groups at the N-terminus of Phe via a simple amide bond, respectively [103]. All the Phe derivatives can form thermally and pH-reversible gels. Interestingly, cinnamoyl-Phe can form cis isomers under UV (254 nm) irradiation accompanied with the formation of both a solid phase and fluid phase. TEM experiments demonstrated that the naphthyl-Phe, naphthalenoxyl-Phe, and cinnamoyl-Phe gels consisted of a fibrous network, while nanoparticles (20–80 nm) were strung together to constitute the matrices of Fmoc-Phe. This morphology of Fmoc-Phe is different from the result reported by Roy and coworkers, who observed fibrous structures [16], owing to the different sample preparation process or condition.

Nanda and colleagues reported a pyrene-modified Phe (Pyr-Phe), which can form a hydrogel over a wide pH range (7.46–14) [104]. However, the gelation failure was observed in the case of Pyr-Val, indicating that the gelation behavior was mainly driven by π–π interactions. The storage modulus of the hydrogel with tape-like nanofibers at a pH > 10.5 is much higher than that of a hydrogel with helical nanofibers at a pH < 10.5. As a result, the resulting hydrogel samples at a pH < 10.5 can transform into a sol phase by shaking or a vortex, instead the hydrogels obtained at a pH > 10.5 did not show any thixotropic property. The pH-dependent morphological difference may arise from the salting-out effect, which promoted the π–π stacking and strengthened the intermolecular interactions as demonstrated by UV-vis and X-ray diffraction data. Taking advantage of the reversible redox property of ferrocene (Fc), a redox-sensitive hydrogel was designed based on self-assembled Fc-Phe nanofibers [105]. The addition of H_2_O_2_ can lead to the degradation of the nanofibers and the collapse of the hydrogel networks owing to the electrostatic repulsion of the cationic Fc. This behavior facilitated the development of a novel electrochemical immunosensing platform for the detection of human IgG with good reproducibility, high specificity, and stability.

### 3.2. Self-Assembly of Alkylated Amino Acid Derivatives

**Figure 15:**
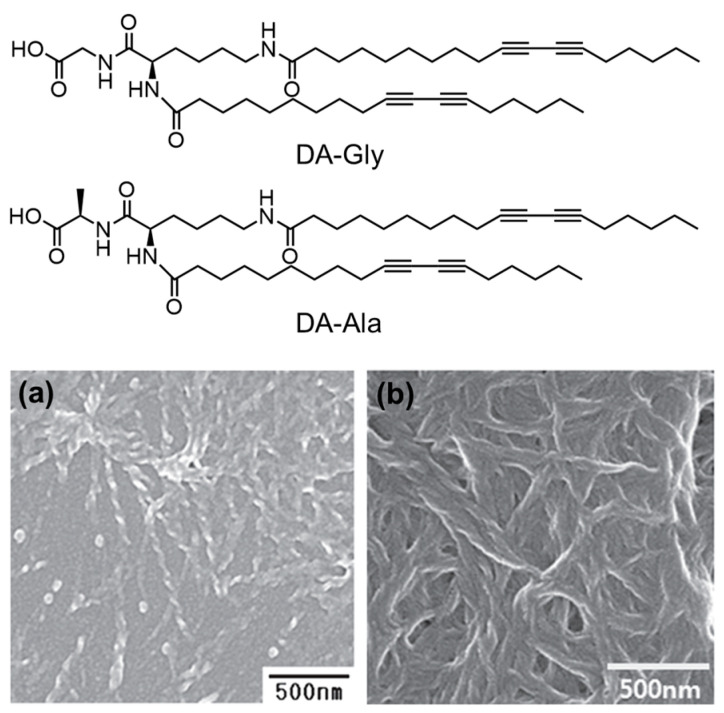
Chemical structures of DA-Gly and DA-Ala and their SEM images: (**a**) DA-Gly, (**b**) DA-Ala. Reproduced with permission from Ref. [108]. Copyright 2011 American Scientific Publishers.

Alkylation is another strategy to promote the assembly of AAs. Mohanty et al. synthesized sodium *N*-(4-dodecyloxybenzoyl)-l-valinate (C_12_BZ-Val) [106]. The interplay of hydrogen bonds, hydrophobic, and π-π interactions enabled the amphiphilic C_12_BZ-Val to form abundant morphologies at different concentrations. For example, spherical vesicles with a bilayer structure were observed at a low concentration, whereas tubules, straps, double helix ropes, and rod-like nanostructures were predominant at relatively higher concentrations. The authors suggested that the tubules and rod-like structures resulted from the up-scrolling of flat lamella. Along a similar line, N-lauroyl-L-glutamic acid (C_12_-Glu) was designed to generate a supramolecular hydrogel, which was utilized as a template for the preparation of CuS nanoribbons [107]. Recently, the realm of alkylated AAs has been extended to diacetylene-modified Gly (DA-Gly) or Ala (DA-Ala), which can self-assemble into a hydrogel with helical fiber networks [108]. As shown in Figure 15, the resulting DA-Gly showed well-defined helical structures with tightly molecular stacking, while loose helical fibers were observed in the case of DA-Ala. The steric hindrance of the methyl residue of Ala may reduce the strength of intermolecular hydrogen bonds, giving rise to the formation of loose molecular stacking. Interestingly, the photo-initiated polymerization of diacetylene can largely improve the heating stability of the hydrogels without changing their inherent morphologies. As a result, a thermochromic hydrogel could be produced in the case of DA-Gly by regulating the molecular stacking between a loose and tight state. However, this thermochromism cannot be observed in DA-Ala, where the existence of steric hindrance prevented tight stacking. The thermochromic hydrogel hold great promise in applications such as surface coating, artificial retinas, neuronal networks, and nanoelectronics.

Patra and coworkers reported a family of pH-sensitive hydrogels based on alkylated His (C_n_-His, n = 6, 8, 10, 12) with different chain lengths, which self-assembled into helical fibers and were further bundled or entangled into 3D network structures through hydrophobic interactions, π-π stacking, and intermolecular H-bonds [109]. The existence of imidazole and α-COOH groups endowed the hydrogel with pH sensitiveness. The authors concluded that the gelation kinetics of C_n_-His was proportional to the amount and chain length of the alkyl chains. In another work, *N*-lithocholyl-(cysteine ethyl ester) (C_24_-Cys) and *N*-lithocholyl-(valine ethyl ester) (C_24_-Val) were synthesized by conjugating bile acids with amino acid esters [110]. By harnessing the self-assembly capacity of the long alkyl chain of the bile acid moiety, C_24_-Cys and C_24_-Val can form either spherical or burl-like 3D aggregates depending on the sample preparation process. Subsequently, the bile acid moiety was further conjugated with aromatic and aliphatic AAs [111], giving rise to the formation of transparent or translucent hydrogels. Besides the long aliphatic chains, short alkyl chains were also conjugated with natural AAs to produce a self-assembling system. As illustrated by Li and colleagues, thixotropic hydrogels with thermally reversible sol–gel transition were prepared via the aqueous self-assembly of menthol methyl ester group-modified Lys [112]. A SEM image revealed that the hydrogel was composed of fibrous three-dimensional networks. The acid-based interaction and the strength of the hydrogen bond between molecules are the key factors for the formation of the hydrogel. In addition, the menthol methyl ester group-modified lysine can co-assemble with some antibacterial agents (such as Zn^2+^, lincomycin, amoxicillin, etc.) in a unique way to function as an antibacterial matrix. The antibacterial sensitivity of the hydrogel loaded with Zn^2+^ or lincomycin was much more effective than that of the antibacterial agents alone. Similarly, a poly(Asp)-based hydrogel with fibrous structures with a diameter of ~1 μm was synthesized [113]. The hydrogels with free carboxyl groups exhibit rapid volume transitions and a high swelling ratio induced by pH, which can be applied to drug delivery and biological tissue engineering.

**Figure 16:**
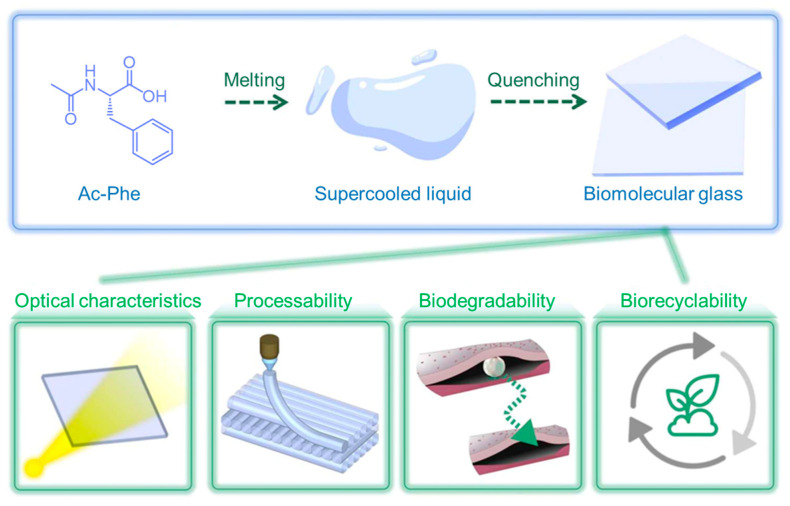
Schematic drawing of biomolecular glass with unique properties resulting from the supercooled Ac-Phe liquid. Reproduced with permission from Ref. [13]. Copyright 2023 American Association for the Advancement of Science.

Cao and colleagues designed Ala derivatives by modifying the N- and C- terminus with Fmoc and a long alkyl chain (Fmoc-Ala-C_17_), respectively [114]. The enantiomeric Fmoc-Ala-C_17_ alone self-assembled into a fibrous nanostructure that transformed into nanoribbons when increasing the aging time in hexane. However, the equimolar-mixed racemate Fmoc-*L*-AlaC_17_ and Fmoc-*D*-AlaC_17_ formed uniform twisted ribbons with a width of about 1 mm and a pitch of more than 5 mm. FT-IR, UV-vis, and circular dichroism spectra identified that the self-assembly behavior was controlled by hydrogen bonding and π–π stacking. In particular, the authors extracted a new form of majority rule in this system in that a slight excess of an enantiomer showed more efficacy in controlling the handedness of the twisted ribbons. This rule enabled the system to be a supramolecular chiral sensor for either discriminating some chiral AA derivatives (Fmoc-Ala-OH) or determining the ee values of the racemic system. Branched Lys derivatives with different topological structures have been synthesized by grafting Lys onto polyhedral oligomeric silsesquioxane (POSS) [115]. The resulting POSS-Lys with a cubic topological core has stronger gelation ability in various solvents. However, the C_12_-Lys with a linear topological core structure shows weak gelation propensity, while pentaerythritol-modified Lys (PER-Lys) cannot form a gel in any solvent owing to the steric hindrance effect of the tetrahedral topological core structure of the PER molecule. These outcomes emphasized the importance of the topological structure of the AA derivatives in the self-assembly and gelation process. Brar et al. investigated the self-assembly of the γ-AA derivative, *t*-butyloxycarbonyl-γ-amino butyric acid (Boc-γ-ABA)-N,N’-dicyclohexylurea [116]. The amphiphilic compound formed dense rod-like clusters in dimethyl sulfoxide (DMSO), overlapping motifs in methanol, clear nanorods in acetonitrile, and sheet-like structures in toluene. It is clear that the polarity of the solvent has a significant influence on the interplay of hydrogen bonds, van der Waals forces, and ionic bonds. Different from the ordered assembly, Xing and coworkers reported a kind of amorphous bioglass via kinetic freezing of the melted liquids of acetyl-amino acids (Ac-AAs) (Figure 16) [13]. The authors demonstrated that the cooperation of multiple weak intermolecular interactions (such as multiple types of hydrogen bonds) facilitated the stabilization of the molecular glass with disordered molecular packing. These glasses exhibited excellent optical characteristics and flexible processability, as well as biodegradability and biorecyclability.

## 4. Co-Assembly of Amino Acid Derivatives and Various Objects

### 4.1. Co-Assembly of Amino Acid Derivatives and Their Counterparts

**Figure 17:**
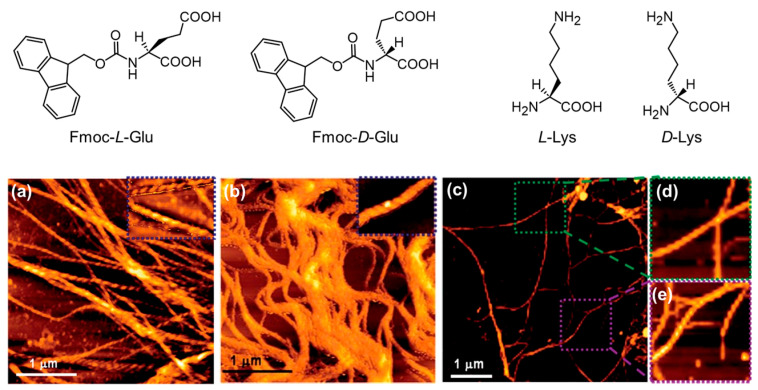
Chemical structures of Fmoc-*L*-Glu, Fmoc-*D*-Glu, *L*-Lys, and *D*-Lys, and the AFM images of the co-assembled nanostructures of Fmoc-*L*-Glu/*L*-Lys (**a**), Fmoc-*D*-Glu/*D*-Lys (**b**), and Fmoc-*L*-Glu/*L*-Lys with Fmoc-*D*-Glu/*D*-Lys (**c**–**e**), where (**d**) and (**e**) are the selected area from (**c**). Reproduced with permission from Ref. [117]. Copyright 2011 Royal Society of Chemistry.

Apart from their self-assembly, AA derivatives can also co-assemble with various objects to generate hierarchical nanostructures and value-added soft materials. Adhikari et al. reported that Fmoc-Glu and oppositely charged alkaline AAs (such as Lys, Arg, and His) can co-assemble into nanofibers, where the chirality of the nanofibers depended on the chirality of individual building blocks [117]. For example, Fmoc-*L*-Glu and *L*-Lys co-assembled into a hydrogel with left-handed helical nanofibers, while right-handed helical nanofibers were observed in the case of a Fmoc-*D*-Glu/*D*-Lys hydrogel (Figure 17). When mixing (Fmoc-*L*-Glu/*L*-Lys) and (Fmoc-*D*-Glu/*D*-Lys) in an aqueous solution, self-sorting behaviors with equal amounts of left- and right-handed helical nanofibers were observed. Similar phenomena were observed by replacing Lys with ornithine (Orn) or Arg. Interestingly, the introduction of divalent metal ions Ca^2+^ or Mg^2+^ can lead to the transformation from helical nanofibers to straight fibers via the non-covalent interactions between metal ions and the carboxylic acid of Fmoc-Glu.

**Figure 18:**
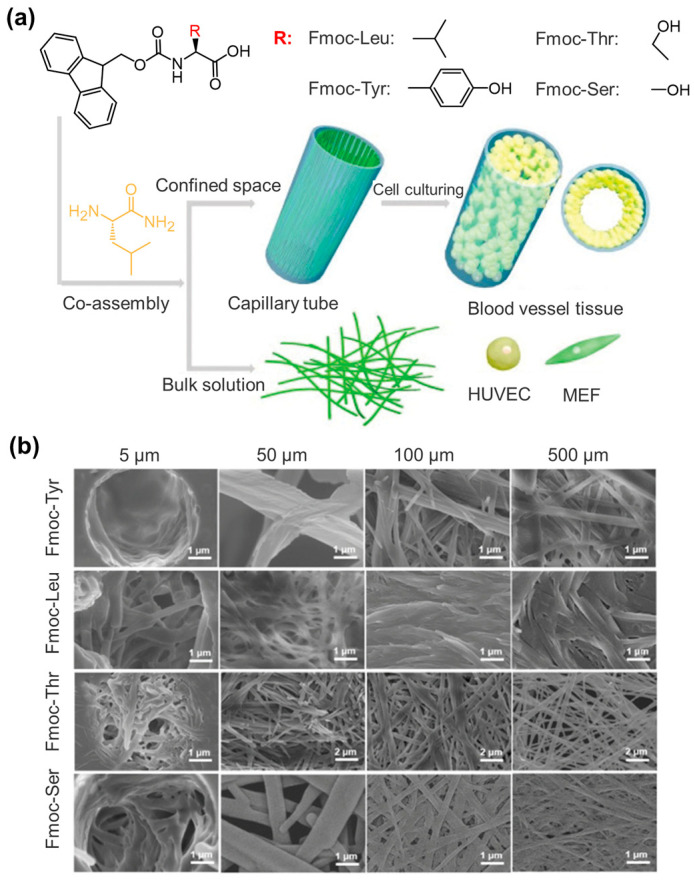
(**a**) Schematic drawing of the confined co-assembly of Leu-NH_2_ and Fmoc-AAs and the model for cell (HUVEC and MEF) adhesion and proliferation; (**b**) SEM images of confined co-assemblies of Leu-NH_2_ with Fmoc-Tyr, Fmoc-Leu, Fmoc-Thr, and Fmoc-Ser, respectively, in capillary with diameters from 5 to 500 μm. Reproduced with permission from Ref. [123]. Copyright 2022 Royal Society of Chemistry.

Fmoc-Leu and Fmoc-Lys can also co-assemble into twisted nanoribbons via π-π stacking and hydrophobic interactions [118]. The nanoribbons can be entangled into hydrogels with broad-spectrum antimicrobial activity against Gram-negative and Gram-positive bacteria. In particular, the mechanical properties and antimicrobial activities of the co-assembled hydrogels could be conveniently adjusted by controlling the molar ratio of the components. This enhanced stability of the co-assembled hydrogel has been observed in different systems. For instance, Fmoc-Phe- and Fmoc-modified Lys at *ε*-NH_2_ (Fmoc-(N^ε^)-Lys) have been utilized to fabricate a stable hydrogel, which can encapsulate luminol and hemeprotein with enhanced chemiluminescence [119]. This co-assembled system can be used for detecting trace blood. A stable hydrogel consisting of densely entangled fibrils with an average diameter of 16 ± 2 nm was obtained by combining the rigidity of the Fmoc-F_5_-Phe fibers with the solvolytic stability of the polyethylene glycol-modified Fmoc-F_5_-Phe (Fmoc-F_5_-Phe-PEG) fibers [120]. In another report, Misra et al. illustrated that Fmoc-γ-Phe self-assembled into a self-healing hydrogel, while Fmoc-(3-hydroxy)-γ-Phe formed a transient hydrogel but subsequently changed into microcrystalline aggregates due to the presence of additional hydroxyl groups. However, the co-assembled Fmoc-γ-Phe/Fmoc-(3-hydroxy)-γ-Phe hydrogel retained the self-healing behavior of Fmoc-γ-Phe but inhibited the phase transition of Fmoc-(3-hydroxy)-γ-Phe, giving rise to the formation of a homogenous and stable hydrogel [121]. Similarly, the co-assembly of naphthalene diimide (NDI)-Ser and NDI-Lys can greatly improve the stability of the hydrogel compared to that of individual building blocks [122]. Dong et al. investigated the confined co-assembly of Fmoc-protected AAs and Leu-NH_2_ carrying a C-amide group [123]. As illustrated in Figure 18, the co-assembled structures gradually transformed from disordered fibers to network structures when increasing the capillary diameter from 5 to 100 μm and beyond. The diameter of the fibers formed in a small diameter capillary tube was larger than that formed in large diameter capillary tubes. In addition, the co-assembled structures of Fmoc-Tyr/Leu-NH_2_ and Fmoc-Leu/Leu-NH_2_ were more ordered than those of Fmoc-Thr/Leu-NH_2_ or Fmoc-Ser/Leu-NH_2_. The authors proposed that the more hydrophilic Fmoc-Thr and Fmoc-Ser may tend to form hydrogen bonds with the capillary channel walls, thereby disrupting the growth of parallel capillary direction fibers. The formed ordered fiber networks, serving as an extracellular matrix, can support the adhesion and proliferation of human umbilical vein endothelial cells (HUVECs) and mouse embryo fibroblast (MEF) cells, ultimately forming a tubular structure similar to blood vessel tissue.

### 4.2. Co-Assembly of Amino Acid Derivatives and Organic Objects

**Figure 19:**
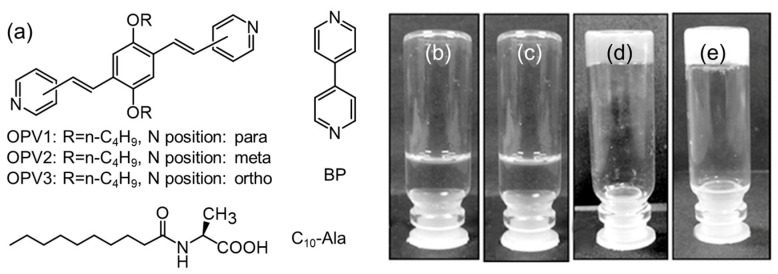
Chemical structures (**a**) of OVPs, BP, and C_10_-Ala, and the photographs of co-assembled samples of C_10_-Ala/OPV3 (**b**), C_10_-Ala/OPV2 (**c**), C_10_-Ala/OPV1 (**d**), and C_10_-Ala/BP (**e**). Schemes follow the same formatting. Reproduced with permission from Ref. [124]. Copyright 2013 Wiley-VCH.

Apart from the AA-based objects, a large amount of organic ligands have been complexed with AA derivatives. Bhattacharjee et al. reported an anisotropic gel via the co-assembly of *N*-Decanoyl-l-alanine (C_10_-Ala) and pyridine-end oligo(p-phenylenevinylene)s (OPVs) [124]. As shown in Figure 19, the alkalinity of the N atom on the pyridine ring in OPVs dominates the gelation behavior via the ionic and hydrogen bonds. Only the OPVs with the N atom located at the para position of the pyridine ring can co-assemble with C_10_-Ala into a stable gel with rectangular plate-like morphologies. A similar phenomenon can be observed in the case of 4,4′-bipyridine (BP). In another work, a discrete propeller-like supramolecule was constructed via the co-assembly of chiral N-octanoyl-amino acids (C_8_-AAs) and achiral hexa-2-pyridyl-hexaazatriphenylene [125]. The sergeants-and-soldiers principle and majority-rules effect have been applied to achieve the chiral amplification [126]. A similar phenomenon was observed in the co-assembly system of Fmoc-AAs and 1,3,5-tri(1H-benzo[d]imidazol-2-yl)benzene [127].

**Figure 20:**
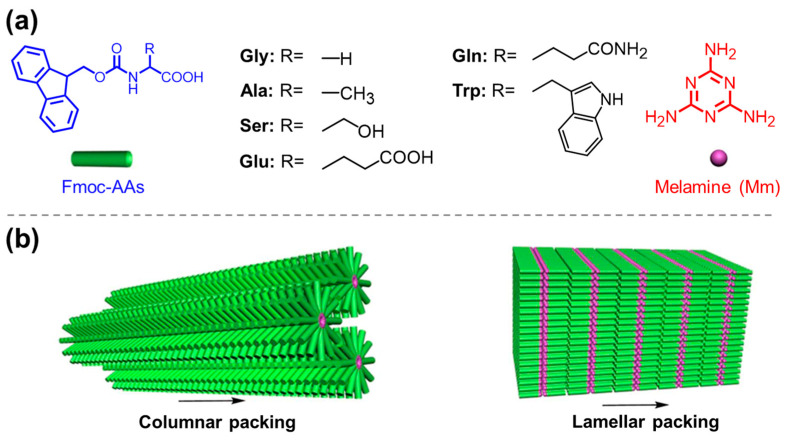
(**a**) Chemical structures of Fmoc-AAs and melamine; (**b**) schematic drawing of the co-assembled columnar and lamellar structures based on Fmoc-AAs and melamine. Reproduced with permission from Ref. [128]. Copyright 2017 American Chemical Society.

Xing et al. investigated the co-assembly behavior of different Fmoc-AAs and melamine (Mm), and found that the residues’ molecular conformation of the Fmoc-AAs can control the assembly pathway [128]. As shown in Figure 20, Fmoc-Gly, Fmoc-Ala, and Fmoc-Ser have a strong propensity to form a lamellar filling pattern and three-dimensional aggregates with Mm, while the other Fmoc-AAs with large residues favored the formation of one-dimensional aggregates (including the hexagonal and quadrangular pattern) with Mm. Based on this finding, the authors fabricated a kind of co-assembled hydrogel based on Fmoc-Phe and 1,2-di(4-pyridyl)ethylene (BPE) [129]. The resulting hydrogel showed enhanced green fluorescence under UV irradiation owing to the *trans*-to-*cis* isomerization of BPE. Similarly, luminescent gels can be conveniently constructed via co-assembly of a perylene-functionalized Phe and 4,4’-bipyridine [130] or the co-assembly of Val derivatives (*N*,*N*’-divaline-3,4,9,10-perylenetetracarboxylic acid), riboflavin, and Mm with the aid of multiple hydrogen bonds and π-π stacking [131].

**Figure 21:**
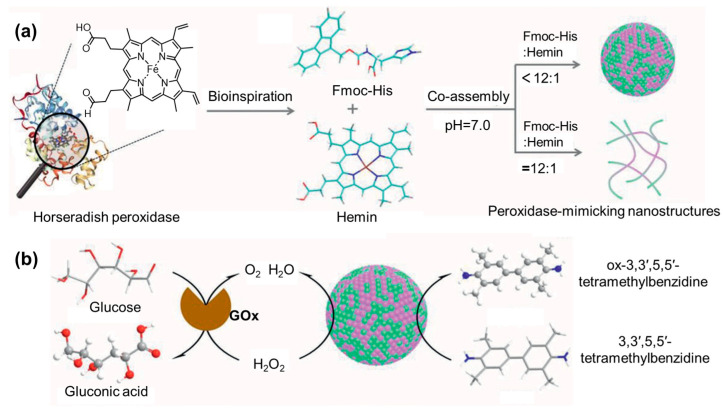
(**a**) Schematic drawing of the co-assembly of Fmoc-His and hemin; (**b**) co-assembled Fmoc-His/hemin nano-enzyme serving as peroxidase for the oxidation of 3,3′,5,5′-tetramethylbenzidine. Reproduced with permission from Ref. [134]. Copyright 2021 Wiley-VCH.

Following the same procedure, a multicomponent hydrogel was prepared based on Fmoc-Tyr, guanosine monophosphate (GMP), and Ag^+^ [132]. It was shown that the mechanical properties of the hydrogel can be flexibly regulated by controlling the molar ratio of Ag^+^ to GMP. When combining AA derivatives with bioactive components, an antimicrobial hydrogel was fabricated via the co-assembly of Fmoc-Phe and the antibiotic aztreonam (AZT). The authors proposed that Fmoc-Phe and AZT had a synergistic antimicrobial effect on, where AZT affected the integrity and permeability of the bacterial cell membrane and increased the uptake of Fmoc-Phe by the bacterial cells, giving rise to broad-spectrum antimicrobial activity [133]. In addition, an AA-based nano-enzyme with peroxidase-mimicking activities has been designed via the co-assembly of Fmoc-His and hemin derivative (Figure 21a) [134]. The optimized co-assemblies showed comparable catalysis activities to those of natural horseradish peroxidase (Figure 21b). Thus, it can be used for the detection of glucose by coupling with glucose oxidase.

Graphene oxide (GO) has also been incorporated with Fmoc-Phe to develop a hybrid hydrogel [135]. It was found that GO has the capacity to modulate the gelation process and the viscoelastic properties of the co-assembled hydrogel. Additionally, a co-assembled Fc-Phe/GO hydrogel has been explored as an electrode coating for detecting dopamine [136]. Furthermore, a conductive hydrogel was prepared via the ionic co-assembly of Fmoc-Phe and polyaniline (PANI) [137]. The Fmoc-Phe/PANI gel exhibited good strain recovery, mechanical strength, and electrical conductivity, and has shown its potential in soft electronics, optoelectronics, and biosensors. The ionic co-assembly between anionic AA surfactants and cationic cellulose enabled the formation of condensed coacervates with a fibrous structure of a few micrometers in diameter [138]. Similar condensation behavior was observed in the case of a mixed micelle system composed of sodium lauroylsarcosinate and amphoteric surfactant cocamidopropyl betaine with cationic guar gum [139]. The electrostatic attraction between guar gum and the mixed micelles drives the formation of a complex coacervate with porous interior. The authors demonstrated that the properties of the coacervate can be easily controlled by adjusting the mixed micelle ratio, salt concentration, and dilution ratio.

### 4.3. Co-Assembly of Amino Acid Derivatives and Inorganic Objects

**Figure 22:**
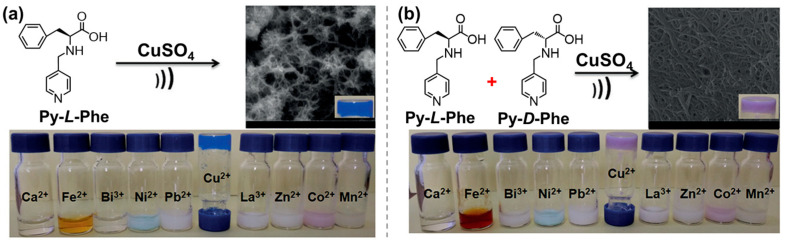
Chemical structures of Py-*L*-Phe (**a**) and Py-*D*-Phe (**b**), and the preparation of Cu^2+^-hydrogels using (**a**) Py-***l***-Phe (0.1 M) or (b) Py-*l*-Phe + Py-*D*-Phe (each of them, 0.05 M) and CuSO_4_ (0.1 M) with a molar ratio of 2:1, by sonication treatment in water. Complex images of Py-*l*-Phe (**a**) or Py-*l*-Phe + Py-*d*-Phe (**b**) and different metal ions under the same conditions are shown at the bottom. Reproduced with permission from Ref. [140]. Copyright 2018 Wiley-VCH.

In fact, many inorganic objects are luminescent, magnetic, redox active, catalytic, photosensitive, or bioactive [72,73]. Co-assembly of AA derivatives and inorganic objects represents a promising method for the development of value-added materials. Pyridine-modified Phe (Py-Phe) can selectively coordinate with copper ion (Figure 22), forming a blue metallo-hydrogel [140]. However, when Py-*L*-Phe and Py-*D*-Phe were mixed in an equimolar ratio, a pink Cu^3+^ superoxide gel [(*L* + *D*)-Py-Phe-Cu^3+^-O_2_^-^] with single-electron O_2_ reduction activity was obtained due to the robbing of one electron of Cu^2+^ by the coordinated O_2_. In another work, imidazole-modified Trp (Im-Trp) was designed to selectively co-assemble with Ni^2+^ for the construction of metallo-hydrogel [141]. Equimolar Im-*L*-Trp and Im-*D*-Trp can coordinate specifically with Ni^2+^ to form purple hydrogels with the aid of π-π stacking and hydrogen bonds.

**Figure 23:**
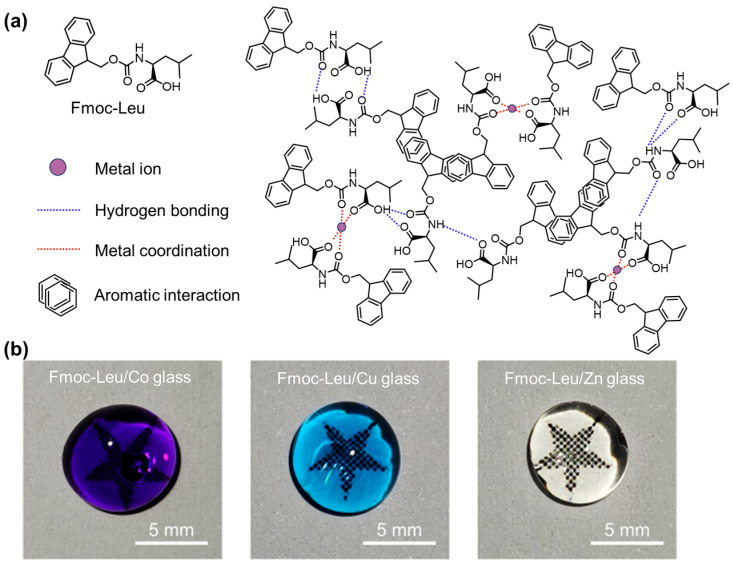
(**a**) Chemical structure and the packing model of Fmoc-Leu; (**b**) photographs of the bioglass resulting from the co-assembly of Fmoc-Leu with Co^2+^, Cu^2+^, and Zn^2+^, respectively. Reproduced with permission from Ref. [147]. Copyright 2024 Chinese Chemical Society.

Sodium deoxycholate (NaDC)-conjugated β-*l*-Phe (β-*l*-PheDC) can co-assemble with Ca^2+^ at pH 12 to form a hydrogel with helical strips [142]. However, morphological transition from helical structures to a flat one was observed when pH decreased to 10 because of the partial protonation of the -NH_2_ group competing with Ca^2+^ for the interaction with the carboxylic group. Ma and coworkers reported a kind of stimuli-responsive metallo-hydrogel based on the co-assembly of Fmoc-Val and Zn^2+^ or Cu^2+^ via hydrogen bonds, coordination bonds, and π-π stacking [143]. The resulting hydrogels, consisting of rod-like microstructures, exhibited multi-stimuli responsiveness against temperature, metal ions, and acids, etc. Incorporating Fmoc-AAs (Fmoc-His, Fmoc-Pro, Fmoc-Ala, Fmoc-Leu, and Fmoc-Phe) and Ag^+^ resulted in antimicrobial hydrogels [144,145]. It was suggested that the increased antimicrobial efficiency could be attributed to the continuous release of Ag^+^ or the synergistic effect of the in situ-formed Ag nanoparticles and amphiphilic Fmoc-AAs. A ternary co-assembly system has been designed by Li and coworkers based on Fmoc-Phe, nano-hydroxyapatite (nHAP), and chlorogenic acid (CGA) [146], leading to the formation of a value-added hydrogel. Fmoc-Phe could self-assemble into a fibrous structure and provide a network matrix, the nHAP bound in the chamber of the fibrous network of Fmoc-Phe significantly improved the mechanical strength of the hydrogel, and the CGA showed a good antibacterial activity against *S. aureus*, giving rise to a strong and antimicrobial hydrogel. In the out of water condition, Fmoc-Leu and metal ions (Co^2+^, Cu^2+^, Zn^2+^) can form bioglass (Figure 23) under the joint drive of hydrogen bonds, metal coordination, and aromatic interactions [147]. The bioglass showed good hardness and wear resistance superior to those of traditional polymers, while also exhibiting elasticity comparable to ceramics. Replacing the metal ions with rare earth ions (Eu^3+^, Tb^3+^) endowed the bioglass with tunable fluorescence emission.

**Figure 24:**
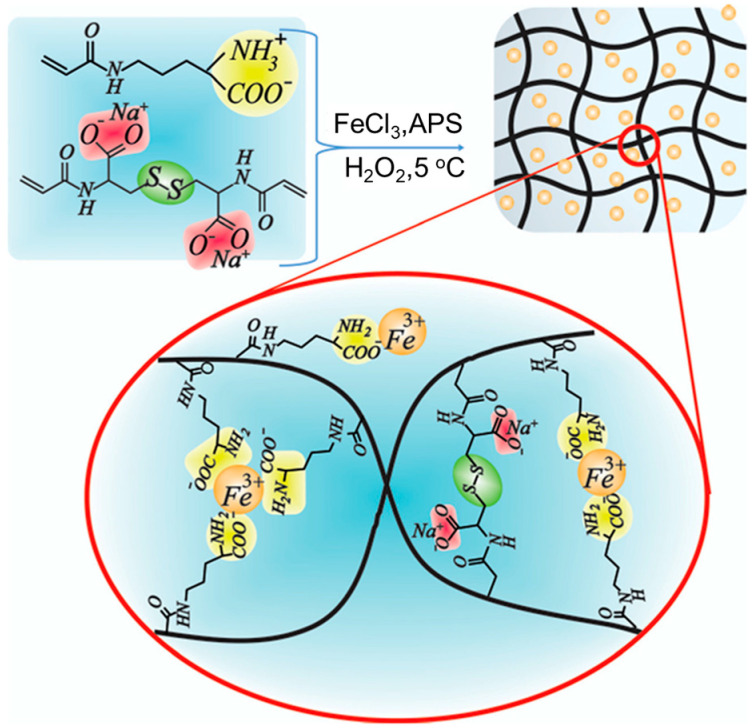
Chemical structures of N-δ-acryloyl ornithine (Ac-Orn) and N-N’-bisacryloylcystine and the co-polymerized hydrogel with the assistance of Fe^3+^. Reproduced with permission from Ref. [149]. Copyright 2022 Royal Society of Chemistry.

Romanski et al. designed a kind of copolymer by free radical polymerization of acryloyl and methacryloyl Orn or Lys derivatives with N-isopropylacrylamide [148]. After co-assembling with Cu^2+^ or Co^2+^, the resulting hydrogel showed significant volume shrinkage owing to the significantly increased crosslinking density. Recently, N-δ-acryloyl ornithine (Ac-Orn) and *N*-*N*’-bisacryloylcystine have been utilized to copolymerize into hydrogel in the existence of Fe^3+^ (Figure 24) [149]. The prepared hydrogel showed good mechanical properties, self-healing ability, and electrical conduction. In addition, the dynamic disulfide bonds enabled the reversible gel-to-sol transformation with the assistance of oxidizing or reducing agents. Block-co-polymer P(AM-HisMA), consisting of His methacrylamide (HisMA) and acrylamide (AM), can form physically crosslinked hydrogels via multiple hydrogen bonds [150]. After coordinating with Fe^3+^, the resulting metallo-hydrogel P(AM-HisMA)/Fe^3+^ exhibited good mechanical properties, tissue adhesion, antimicrobial properties, and low cytotoxicity, showing potential in wound healing. Apart from the metal ions, AA derivatives can also co-assemble with POM to construct an efficient catalyst for the oxidation of aromatic alkenes to the corresponding oxirane [151].

## 5. Conclusions and Prospects

This article provides an overview of AA-derived supramolecular assembly and soft materials. The assembly ability of most natural AAs (except Trp, Tyr, and Phe) is poor, but the conjugation of hydrophobic groups with natural AAs or the co-assembly of AAs and derivatives with other objects provided powerful tools to create various structures and soft materials through the nano- and micro-scales up to the macroscopic level, including vesicles, particles, rods, nanofibers, helix, ribbons, tubules, sheets, flakes and plates, nanoflowers, hedgehog superstructures, networks, hydrogels, complex coacervates, and amorphous bioglass (Table 1). The various assembling structures are beginning to show great potential in sustained drug release with reduced toxicity, antimicrobial, antiviral, and antitumor activity, cell adhesion, wound dressing, tissue engineering, diagnostics, biomimetic underwater adhesion, biosensors and detectors, coatings, bioglass, chiroptical materials, catalysis, electrochemistry, and food chemistry. Systematic studies show that the assembled process is driven by combined interactions, such as hydrogen bonds, ionic bonds, coordinate bonds, hydrophobic effects, and CH-π, and π-π stacking [29,44,79,107], while, ultimately, the assembled morphologies are dependent on the relative volume fraction of hydrophobic and hydrophilic blocks, the molecular geometry and conformation, and the kinetic pathway. Although the knowledge of how the non-covalent interactions can be judiciously harnessed to exert the ordered assembly has been collected, ongoing efforts are still necessary to fully understand the entire assembly pathway with the aim of developing tailor-made nanostructures and materials. In many cases, the resulting assemblies showed kinetically labile and dynamic features, which enables these systems to be stimuli-responsive. It is worth highlighting that the modular co-assembly between AAs/derivatives and other objects is an attractive and effective strategy for the creation of value-added soft materials without any time-consuming and inaccessible chemical synthesis. Therefore, the minimalistic and flexible co-assembly may provide infinite possibilities in the creation of AA-based nanostructures and supramolecular materials with many applications beyond the current body of scientific literature. Although, the AA-derived supramolecular assembly remains exciting, and interest in the soft materials continues to grow, more efforts are still needed to scrutinize the relationship between the hierarchical structure of AAs and their material properties. It is expected that more consideration may be given to the chiral nanostructures, environment, and properties of AA assemblies.

## Figures and Tables

**Table 1:** Summarization of AAs and derivates and the assembled structures.

Compounds	Assembled Structures
Phe/Phe@Au or Cit@Au [15]	nanofibers nanoribbons
His/Cit@Au [15]	
Phe/Zn^2+^ or Cd^2+^ or Hg^2+^ or Al^3+^ [48]	
Fmoc-His [97]	
Fmoc-Ala-C_17_ [114]	
Fmoc-Tyr or Fmoc-Thr or Fmoc-Ser or Fmoc-Leu/Leu-NH_2_[123]	
Phe or Trp [31]	
Phe/Tyr/Trp [33]	
Tyr [26]	
His/Phe@Au [15]	dendritic structures
Trp/Phe@Au or Cit@Au [15]	
Try or Trp [27]	nanotubes
(Ile)_3_PMo_12_O_40_ [72]	
(Ile)_3_PW_12_O_40_ [72]	
(Cys)_3_PMo_12_O_40_ [72]	
(Cys)_3_PW_12_O_40_ [72]	
*L*-Phe [28]	hydrogel with fibrillar structures hydrogel with twisted nanoribbons hydrogel with rod-like structures
Fmoc-Tyr [85,86,93]	
Fmoc-Met [93,94,95]	
Fmoc-Phe [17,88]	
Fmoc-Trp [93]	
Fmoc-β-Phe [92]	
N- and C-terminus of Asp were modified by Fmoc [96]	
Fmoc-Met or Fmoc-Gly or Fmoc-Ile [94]	
Cbz-Phe [99,100]	
Fc-Phe [105]	
DA-Gly or DA-Ala [108]	
Cn-His [109]	
β-*L*-PheDC/Ca^2+^ [142]	
menthol methyl ester group-modified Lys [112]	
Fmoc-Glu/Lys [117]	
Fmoc-F5-Phe/Fmoc-F5-Phe-PEG [120]	
Fmoc-Leu/Fmoc-Lys [118]	
L-Phe/Zn^2+^ [46]	
Phe [29]	hydrogels
Lys or Glu/chitosan/αβ-glycerophosphate [40]	
β-Ala/chitosan/poly-(γ-glutamic acid) [41]	
Cys/silver nitrate [14,42,43]	
Fmoc-F_5_-Phe [91]	
Cbz-Trp [101]	
N-(4-nitrobenzoyl)-Phe [102]	
naphthyl-Phe [103]	
naphthalenoxyl-Phe [103]	
cinnamoyl-Phe [103]	
Pyr-Phe [104]	
C_12_-Glu [107]	
POSS-Lys or C_12_-Lys [115]	
Fmoc-Phe/Fmoc-(N^ε^)-Lys [119]	
Fmoc-γ-Phe/Fmoc-(3-hydroxy)-γ-Phe [121]	
(NDI)-Ser/NDI-Lys [122]	
Fmoc-Phe/BPE [129]	
Fmoc-Tyr/GMP/Ag^+^ [132]	
Fmoc-Phe/AZT [133]	
Fmoc-Phe/GO [135]	
Fc-Phe/GO [136]	
Fmoc-Phe/PNAI [137]	
Fmoc-His or Fmoc-Pro or Fmoc-Ala or Fmoc-Leu/Ag^+^ [144]	
Fmoc-Phe/Ag^+^ [145]	
Fmoc-Phe/nHAP/CGA [146]	
Ac-Orn/N-N’-bisacryloylcystine/Fe^3+^ [149]	
DL-Phe [30]	2D plate-like structures
*DL*-mixed Phe or Trp [31]	
Phe/Met [32]	
Phe/Co^2+^ and Pb^2+^ [49]	
Fmoc-Nphe [89]	
Phe/Ile [32] Carboxyl-protected (methyl ester) Phe/[PW_11_O_39_{Sn(C_6_H_4_)CuC(C_6_H_4_)COOH}]^4-^ [60] C_24_-Cys or C_24_-Val [110]	spherical nanostructures
TA/Lys or Arg or His [12]	adhesive with crosslinked networks
His/Zn^2+^ [44]	metallo-hydrogel with nanofiber structures
Phe/Cu^2+^ [45]	
Py-Phe/Cu^2+^ [140]	metallo-hydrogel with sheet-like structures
Im-Trp/Ni^2+^ [141]	
Fmoc-Val/Zn^2+^ or Cu^2+^ [143]	metallo-hydrogel
Phe/Ga^3+^ or In^3+^ [48]	vesicular structures
Arg or Lys or His or Glu or Asp or Leu or Ala or Phe/[EuW_10_O_36_]^9-^ [73]	
Fmoc-Phe/Ag^+^ [16]	
C_12_BZ-Val [106]	
Phe/Cd^2+^ or Zn^2+^ [49]	needle-like structures
Gly or Pro/Cu^2+^/[BW_12_O_40_]^5-^ [61]	three-dimensional open frameworks
His/H_4_SiW_12_O_40_ (SiW) [79]	coacervate
C_12_BZ-Val [106]	tubules, straps, double helix ropes, and rod-like nanostructures
Fmoc-*L*-Ala-C_17_/Fmoc-*D*-Ala-C_17_ [114]	twisted ribbons
perylene-functionalized Phe/4,4’-bipyridine [130]	luminescent gels
N,N’-divaline-3,4,9,10-perylenetetracarboxylic acid/riboflavin/Mm [131]	
Fmoc-Leu/Co^2+^ or Cu^2+^ or Zn^2+^ [147]	bioglass with amorphous structures

## Data Availability

All the data are shown in the manuscript.

## Abbreviations

AAs: amino acids; Tyr: tyrosine; Phe: phenylalanine; Trp: tryptophan; Ile: isoleucine; Met: methionine; Gly: glycine; Ala: alanine; Lys: lysine; Arg: arginine; His: histidine; Glu: glutamic acid; Cys: cysteine; Leu: leucine; Pro: proline; Asp: aspartic acid; Orn: ornithine; Fmoc-Tyr: 9-fluorenylmethyloxycarbonyl-modified Tyr; Fmoc-p-Tyr: phospholated Fmoc-Tyr; Fmoc-Met: Fmoc-modified methionine; Fmoc-NIe: Fmoc-modified norleucine; Cbz-Met: benzoyloxy carbonyl methionine; Cbz-Trp: *N*-[(phenyl methoxy) carbonyl]-*L*-tryptophan; Pyr-Phe: pyrene-modified Phe; Py-Phe: pyridine-modified Phe; Im-Trp: imidazole-modified Trp; Fc: ferrocene; Ac-AAs: acetyl-amino acids; Ac-Orn: N-δ-acryloyl ornithine; HisMA: His methacrylamide; C_10_-AAs: *N*-octanoyl-amino acids; C_10_-Ala: *N*-Decanoyl-*L*-alanine; C_12_BZ-Val: sodium *N*-(4-dodecyloxybenzoyl)-L-valinate; C_12_-Glu: *N*-lauroyl-*L*-glutamic; Cn-His: alkylated His; C_24_-Cys: N-lithocholyl-(cysteine ethyl ester); C_24_-Val: *N*-lithocholyl-(valine ethyl ester); PER-Lys: pentaerythritol-modified Lys; Boc-γ-ABA: t-butyloxycarbonyl-γ-amino butyric acid; β-*L*-PheDC: sodium deoxycholate conjugated β-*L*-Phe; NAAPD: *N*,*N*′-diaspartic acid-3,4,9,10-tetracarboxylic diimide; NDI: diimide; NMD: niclosamide derivative; OPVs: pyridine-end oligo(p-phenylenevinylene)s; Mm: melamine; Mal: malonic acid; NaDC: sodium deoxycholate; BPE: 1,2-di(4-pyridyl)ethylene; GMP: guanosine monophosphate; PANI: polyaniline; AZT: antibiotic aztreonam; GO: graphene oxide; Azo: azobenzene; TDAP: thiophene-2,5-dicarboxamide; nHAP: nano-hydroxyapatite; CGA: chlorogenic acid; AM: acrylamide; AgNPs: silver nanoparticles; POMs: polyoxometalates; HPAs: heteropolyacids; POSS: polyhedral oligomeric silsesquioxane; LLPS: liquid–liquid phase separation; UCST: upper critical solution temperature; ee%: enantiomeric excess percentage; 0D: zero-dimensional; 1D: one-dimensional; 2D: two-dimensional 3D: three-dimensional; *E. coli*: *Escherichia coli*; *S. aureus: Staphylococcus aureus*; TEM: transmission electron microscopy; FE-SEM: field emission scanning electron microscopy; FT-IR: fourier transform infrared; UV-vis: ultraviolet–visible.
